# Chlorophyllin-Mediated Photodynamic Inactivation: Dosage and Time Dependency in the Inhibition of *Bacillus subtilis*

**DOI:** 10.3390/microorganisms13061189

**Published:** 2025-05-23

**Authors:** Sarah Pohland, Jana Vourvoutsiotou, Leah Brandtner, David Geißler, Selina Wiesmeth, Vanessa Scudlo, Peter Richter, Andreas Burkovski, Michael Lebert

**Affiliations:** 1 Cell Biology Division, Department of Biology, Friedrich-Alexander-Universität Erlangen-Nürnberg, 91058 Erlangen, Germany; sarah.pohland@posteo.de (S.P.); jana.vourvoutsiotou@fau.de (J.V.); leah.brandtner@fau.de (L.B.); david.geissler@fau.de (D.G.); selina.wiesmeth@fau.de (S.W.); vanessa.scudlo@fau.de (V.S.); michael.lebert@fau.de (M.L.); 2 Microbiology Division, Department of Biology, Friedrich-Alexander-Universität Erlangen-Nürnberg, 91058 Erlangen, Germany; andreas.burkovski@fau.de; 3 Space Biology Unlimited, SAS, 24 Cours de l’Intendance, 33000 Bordeaux, France

**Keywords:** photodynamic inactivation (PDI), photodynamic therapy (PDT), photosensitizer, chlorophyllin, antibiotic alternatives

## Abstract

Photodynamic inactivation of bacteria offers a promising alternative to counteract the trend towards the development of resistance, which, if left uncontrolled, will lead to the death of 10 million people per year by 2050. Its advantage over antibiotics is the site-specific mode of action due to the photosensitizer (PS) and the low risk of developing resistance. This is primarily prevented by the damage of the bacteria, which also destroy internal structures such as nucleic acid, proteins, and lipids. A promising and still little-researched PS is chlorophyllin (CHL), a chlorophyll derivative. This study investigated its mode of action on *Bacillus subtilis* growth using optical density (OD) measurements. It was shown that the PS is highly effective even at low concentrations and short irradiation durations. Here, 1 mg/L and an irradiation duration of 1 min were sufficient to inhibit the growth of the Gram-positive bacterium *Bacillus subtilis* for several hours.

## 1. Introduction

The environmental microbiome offers a highly diverse gene pool that pathogens can exploit by taking up foreign DNA, thereby developing resistance to antibiotics [1]. This phenomenon, along with other factors—such as mutations in the bacterial genome, overuse in agriculture, or the misuse of antibiotics for viral infections—has led to a situation in which all classes of antibiotics approved to date exhibit resistance in some of the pathogens they are intended to combat [1,2]. If new drugs fail to counteract this trend, it is estimated that 10 million people could die each year from antimicrobial resistance by 2050 [3].

Photodynamic therapy (PDT), or photodynamic inactivation (PDI), offers an alternative approach to treating bacterial infections [4]. Photodynamics relies on the synergistic action of three non-toxic components: a photosensitizer, light at a specific wavelength corresponding to the PS’s absorption spectrum, and molecular oxygen [5]. When the PS absorbs photons, it is excited from its singlet ground state (S_0_) to higher excited singlet states (S_1_, S_2_, and S_3_) [4]. These excited states last only a few nanoseconds before relaxing, and through intersystem crossing, the PS reaches the long-lived triplet state (T_1_). This T_1_ state is responsible for ROS production via electron and energy transfer, which is the cause of the therapeutic effect [4]. In the type I reaction, electron transfer results in the donation of hydrogen atoms or electrons to organic molecules that react with molecular oxygen to form ROS such as O_2_•^−^, •OH, or H_2_O_2_ [6]. This leads to the formation of lipid peroxides and an associated increase in membrane permeability. In the type II reaction, energy transfer produces highly reactive oxygen in the S_1_ state, which damages biological molecules such as DNA, proteins, and lipids through oxidative stress [5].

A well-known porphyrin-base photosensitizer is Chlorophyllin, a water-soluble derivative of the natural plant pigment chlorophyll. Its simple extraction from fresh or frozen plant material makes it an inexpensive option, and its stable chemical structure enables experiments under visible light [7]. Moreover, its high water solubility and absorption maximum at 653.5 nm render chlorophyllin highly suitable for PDT [6].

Concerning reactive oxygen species, these molecules are generated as by-products of cellular metabolic pathways and typically occur at physiological levels; only at high concentrations do they become harmful [8]. Hydrogen peroxide as a representative of ROS, for example, acts as a second messenger in important biological processes and plays a role in morphological changes, proliferation, NF-κB pathway signaling, and apoptosis [7]. An excess of ROS—often as a result of viral infections—can overwhelm the antioxidative defense system and lead to oxidative stress. Proteins, particularly those containing sulfur-rich amino acids like cysteine and methionine, are vulnerable to ROS attack, and oxidative stress can further lead to carbonylation, destabilizing and deactivating bacterial components [9].

To counteract oxidative stress, many organisms produce enzymes such as catalases, peroxiredoxins, and superoxide dismutases, which neutralize harmful ROS [9]. Glutathione peroxidase (GPX) is one such enzyme that comprises several isoenzymes within the glutathione peroxidase family [10]. GPX functions as a homotetramer with selenium at its active center, which is reversibly oxidized during catalysis. It reduces various organic peroxides to alcohols by oxidizing glutathione [11]. In our experiments, GPX activity is measured indirectly through a coupled reaction with glutathione reductase and NADPH, where the oxidation of NADPH to NADP^+^ is monitored as a decrease in absorption at 340 nm. Additionally, the dismutation of hydrogen peroxide into water and oxygen—catalyzed by catalase (CAT)—plays a vital role in protecting cells from oxidative damage. Three types of catalases can be distinguished: the widespread monofunctional form containing heme, a less common bifunctional type also containing heme (closely related to plant peroxidases), and a third type that contains manganese. In the first step of the catalase reaction, H_2_O_2_ is oxidized by heme to form an oxoferrylporphyrin cation radical, which then reacts with a second H_2_O_2_ molecule to produce water and oxygen in a redox process [7,12].

In bacteria, photodynamic reactions lead to the formation of reactive oxygen species (ROS), which damage bacterial cells. Due to the short time between the treatment with photosensitizer (PS) and the application of PDT, as well as the destruction of the outer membrane and structures such as nucleic acid, proteins, and lipids, the formation of resistance is unlikely [4,13]. Gram-positive bacteria are particularly sensitive to inactivation by photosensitizers [14], whereas Gram-negative bacteria exhibit lower sensitivity due to their less permeable outer membrane—a barrier that can be overcome by adding agents such as KI, CaCl_2_, or Tris-EDTA [14,15].

The ubiquitously occurring bacterium *Bacillus subtilis* is the best-studied model for Gram-positive organisms. Under adverse conditions, *B. subtilis* forms endospores, a survival strategy rendering it metabolically inactive and highly resilient; optimal heat activation of these spores occurs between 50 and 65 °C, above which the spore apparatus can be damaged [16,17]. Sporulation in *B. subtilis* is a complex, heterogeneous process controlled by the transcription factor Spo0A, which regulates biofilm production at low concentrations and spore formation at high concentrations [18]. Although the detailed morphological changes during sporulation are not the focus of our study, the ability of *B. subtilis* to switch between vegetative growth and dormancy underscores its value as a model organism.

Beyond sporulation, *B. subtilis* exhibits a sophisticated SOS response involving over 60 genes dedicated to DNA repair and error-free replication [19]. In our study, we examine the expression of key SOS genes—*recA*, *uvrA*, and *mfd*—which are crucial for repairing oxidative DNA damage. The *mfd* gene product initiates transcription-coupled repair, particularly in response to UV-induced large lesions, by coupling lesion recognition to RNA polymerase activity via the nucleotide excision repair (NER) pathway [20]. After RNA polymerase binding, Mfd recruits the UvrAB_2_ complex, which facilitates the separation of the template strand and subsequently recruits UvrC to excise the damaged segment, a gap that is then filled by DNA polymerase I [21]. Meanwhile, *recA*, a central component of the SOS regulon, mediates the transcriptional response to DNA-damaging agents in conjunction with its repressor LexA [19].

To date, only a few studies have examined the photodynamic inactivation of bacteria by chlorophyllin. Most existing work focuses on its effects using high concentrations and long exposure times. In contrast, our study aims to investigate the mode of action of chlorophyllin at low concentrations and with short irradiation durations to assess both its potential efficacy and limitations. By using lower chlorophyllin concentrations and shorter irradiation times, we aim to (i) determine whether these milder conditions can still significantly inhibit bacterial growth and (ii) investigate how the SOS response genes respond under such conditions. This approach offers insight into whether *B. subtilis* can mount a sufficient defense via its DNA repair pathways or if chlorophyllin-based photodynamic treatment overwhelms these protective mechanisms. The findings are expected to inform practical applications of chlorophyllin-mediated PDT, where minimizing light exposure while retaining robust bactericidal activity is often a critical goal.

## 2. Materials and Methods

### 2.1. Phosphate-Buffered Saline (PBS)

Phosphate-buffered saline (PBS; pH 7.4) was prepared with the following composition: 8.0 g/L sodium chloride (NaCl), 0.2 g/L potassium chloride (KCl), 1.44 g/L disodium hydrogen phosphate (Na_2_HPO_4_), and 0.24 g/L potassium dihydrogen phosphate (KH_2_PO_4_) in ultrapure water. The solution was sterilized by autoclaving at 121 °C for 20 min.

### 2.2. Bacterial Strains and Cell Culture

All experiments were carried out with *Bacillus subtilis* strain 168 (laboratory stock, ATCC 23857). Cultures were grown overnight (16–18 h) at 37 °C in Luria–Bertani (LB) medium, composed of 10 g/L tryptone, 10 g/L NaCl, and 5 g/L yeast extract, in ultrapure water. Optical density was determined at 600 nm using a photometer (Ultrospec 2100 pro, Amersham Biosciences, Amersham, UK). For the experiments, overnight cultures were diluted in fresh LB medium and incubated until the exponential phase.

### 2.3. Illumination of B. subtilis

**Figure 1:**
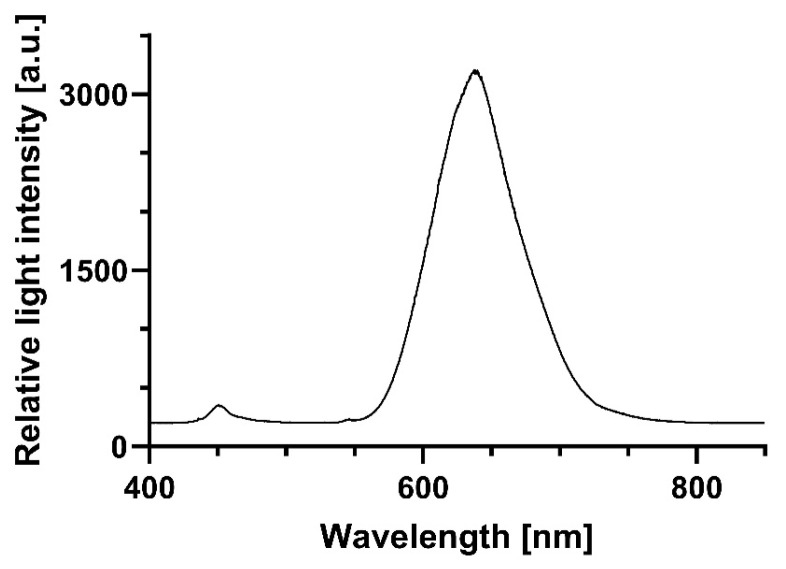
Emission spectrum of red light source for irradiation of *B. subtilis*.

Illumination experiments were performed using a red LED light source (Shenzhen Cooleeon Electronics Co., Ltd., Shenzen; China). The light intensity was 899 µmol photons·m^−2^·s^−1^, equivalent to a radiation of approximately 295.5 W·m^−2^. The total dose cells perceived during 1 min of irradiation was 17.73 kJ/m^2^, 35.47 kJ/m^2^ in 2 min, or 266.95 kJ/m^2^ in 15 min, respectively. The emission spectrum of the red light source is shown in Figure 1.

### 2.4. Chlorophyll Isolation and Transformation into Chlorophyllin

Chlorophyll was extracted from fresh spinach leaves using 100% methanol (VWR International, Radnor, Basking Ridge, NJ, USA) as previously described by Akif et al. (2022) [22]. Chlorophyll concentration was determined according to the method of Ziegler and Egle [23]. Absorption spectra were recorded using the UV/VIS spectrophotometer (UV-2550, Shimadzu, Kyōto; Japan). No interfering substances with absorbance in the ultraviolet or visible light range were detected.

Saponification and transformation to chlorophyllin were performed following the protocol of Krüger and Richter [24], using 100 mM methanolic potassium hydroxide (AppliChem, Darmstadt; Germany). The resulting stock solution was stored at −20 °C in the dark.

For preparation of the working solution, the chlorophyllin extract was evaporated at 45 °C using a vacuum centrifuge (Concentrator 5301, Eppendorf, Hamburg, Germany). The dried residue was reconstituted in LB and subsequently sterile-filtered through a 0.2 µm membrane filter.

### 2.5. Treatment of Bacterial Strains with CHL and Measurement of Optical Density (OD)

Overnight cultures of *B. subtilis* were diluted in LB Medium to an optical density at 600 nm (OD_600_) of 0.05 and incubated at 37 °C with shaking until they reached an OD_600_ of 0.2. The culture was then centrifuged at 418.88 rad/s for 5 min at 4 °C using the Heraeus Biofuge centrifuge (Thermo Fisher Scientific, Waltham, MA, USA). The supernatant was discarded and the cell pellet was resuspended either in fresh LB medium or CHL-conditioned medium. A final concentration of 1 mg/L CHL was used for *B. subtilis*.

Each sample was transferred to a 60 × 15 mm Petri dish with a volume of 5 mL. Samples intended for dark incubation were wrapped in aluminum foil to prevent light exposure. All samples were incubated at 28 °C under a red light source for defined durations (1 min and 15 min). The contents were then transferred to centrifuge tubes and centrifuged under the same conditions. The pellets were washed once with PBS and resuspended in fresh LB medium.

For optical density measurement, 500 µL/well were added in triplicates to a 48-well plate, including the negative control (LB medium only). The samples were incubated at 37 °C and OD_600_ was measured every 20 min using a plate reader (Infinite 200 PRO, TECAN, Männedorf, Switzerland). Prior to each measurement, the plate was shaken linearly for 10 s with an amplitude setting of 6 and a frequency of 295.5 rpm.

### 2.6. Data Evaluation

Optical density (OD_600_) measurements were corrected by subtracting the blank value (LB medium) for both spectrophotometer and plate reader data. All growth experiments were performed in triplicate, with three technical replicates of each condition per run. The mean values of the technical replicates were used, which also served as the basis for the standard deviation. All measurement data were included in the statistical evaluation.

### 2.7. Induction of B. subtilis Spores and Preparation of Spores-Containing Medium for Experiments

Formation of spores was induced by 48 h incubation of 50 mL *B. subtilis* batch cultures on a rotatory shaker. To prepare spore-containing medium, the spent culture medium was harvested by centrifugation (1500× *g*, 5 min, 4 °C) and used to dilute to an OD_600_ of 0.2 (final volume 35 mL). Employing spent medium prevented the spores from germination. To eliminate vegetative cells, spore suspensions were subjected to heat treatment.

### 2.8. Determination of B. subtilis Spores in Overnight Cultures

To confirm the presence of spores in overnight cultures, 500 µL aliquots were heated at 60 °C for 5 min and plated on LB agar. After 24 h of incubation at 37 °C, colony-forming units (CFUs) were counted. Non-heated controls were plated in parallel to differentiate between vegetative cells and spores.

### 2.9. Determination of Effects of CHL-Exposure on B. subtilis Spores

Determination of the effects of CHL exposure on *B. subtilis* spores closely followed the protocol used for vegetative cells. Spores obtained via heat treatment were centrifuged, and part of the supernatant was replaced with an 80 mg/L CHL stock solution in 0.85% NaCl, yielding a final concentration of 1 mg/L CHL. In control samples, an equal volume of 0.85% NaCl solution without CHL was used. Incubation was conducted for 1 min at 28 °C with shaking (light dose: 0.14 µmol/m^2^). Dark controls were protected from light using aluminum foil.

After incubation, cells were washed with 5 mL PBS (supernatant was confirmed spectrophotometrically to be free from CHL traces) and resuspended in 5 mL fresh LB medium. Subsequently, 100 µL aliquots of each sample including the cell-free medium control were plated on LB agar for CFU determination, after 16–18 h of incubation. In addition, samples were transferred to a 24-well microplate (each sample in quadruplicate) and growth was determined using a plate reader (Infinite 200 PRO, TECAN, Männedorf, Switzerland) for 20 h at 37 °C with a sample interval of 20 min.

Furthermore, the effect of light-activated Chlorophyllin on *B. subtilis* was further investigated in additional experiments with lower concentration. Cell pellets from heated spore cultures were obtained after centrifugation, and part of the supernatant was replaced with CHL stock solution to achieve a final concentration of 1 mg/L. Control samples received 0.85% NaCl without CHL. Illumination was again performed for 1 min (0.14 µmol/m^2^), with dark controls wrapped in aluminum foil. Cells were washed with 2 mL LB medium and subsequently resuspended in 1.5 mL fresh LB medium. Different volumes were plated for CFU determination, and parallel aliquots were analyzed for growth in a microplate reader over several hours at 30 min intervals.

### 2.10. Determination of CHL-Induced Transcription Changes in ROS-Relevant Genes in B. subtilis by Means of qPCR

#### 2.10.1. RNA Isolation and cDNA Synthesis

All steps were performed on ice if not otherwise indicated. *B. subtilis* cells (2 mL) were centrifuged (4000× *g*, 5 min) and washed twice with nuclease-free water (7500× *g*, 1 min). The pellet was resuspended in 1 mL of Trizol™ (Invitrogen, Carlsbad, CA, USA) reagent. After 5 min of incubation at room temperature, 200 µL chloroform were added, mixed by vortexing for 15 s, and incubated for 3 min at room temperature. After incubation, the samples were centrifuged (12,000× *g*, 4 °C, 5 min), which resulted in the formation of three distinct layers. Approximately 400 µL of the upper layer containing the RNA was transferred to a new tube, combined with 450 µL ice-cold isopropanol, mixed gently, and incubated for 5 min at room temperature.

After harvesting the precipitated RNA (12,000× *g*, 4 °C, 10 min), the pellet was washed with 1 mL of 75% EtOH (7500× g, 4 °C, 5 min), dried using a vacuum concentrator (Concentrator 5301, Eppendorf, Hamburg; Germany), and resuspended in 20 µL nuclease-free water (storage at −20 °C prior use). For cDNA synthesis, 1 µg of each sample was reverse-transcribed into cDNA using the QuantiTect Reverse Transcription Kit (Qiagen, Venlo; Niederlande) according to the suppliers’ instructions.

#### 2.10.2. qPCR Analysis

Primers were designed using Primer3 software version 4.1.0 (Untergasser et al. 2012) [25]. The following genes were chosen for quantitative analysis: *recA* (transcription increase upon DNA damage response), *uvrA* and *mfd* (nucleotide excision repair), and *gyrA* (housekeeping gene). Gene-specific primer sequences are listed in Table 1.

All primers were initially validated using PCR with PCRBIO VeriFi™ Mix Red (PCR Biosystems, London, UK). Reaction mixtures (20.5 µL total volume) contained 12.5 µL PCRBIO VeriFi™ Mix Red, 1 µL each of forward and reverse primers, and 5 µL cDNA. PCR products were analyzed by agarose gel electrophoresis to confirm specificity. Only the target genes were amplified and no unspecific products were detected.

Primer efficiency was tested by means of dilution series, which were subjected to qPCR. Each qPCR reaction had a final volume of 10 µL, consisting of 5 µL iTaq™ Universal SYBR^®^ Green Supermix (2×, Bio-Rad, Hercules, CA, USA), 0.5 µL of each primer (forward and reverse), and 4 µL of diluted cDNA. Reactions were conducted in 96-well plates using a C1000 Touch™ Thermal Cycler (Bio-Rad). Steps of qPCR were as follows: initial denaturation 95 °C, 1 min; 30 cycles: denaturation 95 °C, 15 s, hybridization 60 °C, 15 s, elongation 72 °C, 15 s; final elongation 72 °C, 2 min. Primer efficiency was determined as follows: *gyrA* 194%, *recA* 111%, *uvrA* 127%, and *mfd* 128%.

Treated samples were analyzed using the same qPCR protocol, except that 4 µL of experimental cDNA was used in place of diluted PCR product. Relative gene expression was calculated using the ∆∆C_t_-Method, employing *gyrA* as a reference gene. Expression levels of *recA*, *uvrA*, and *mfd* were compared to untreated controls (*B. subtilis* grown in LB medium). Cells treated with 150 µM H_2_O_2_ served as a positive control for oxidative stress induction.

Experimental samples were exposed to 1 mg/L CHL. Light-exposed samples received 5 s of red light illumination (15.3 µmol photons·m^−2^·s^−1^), while dark samples were shielded from light. Following treatment, cells were washed with PBS, resuspended in fresh LB medium, and incubated for 30 min to allow gene expression prior to RNA extraction and cDNA synthesis.

### 2.11. Indirect Measurement of Glutathion Peroxidase (GPX) Activity

Prior measurement sample and control cells were harvested by centrifugation (4000× *g*, 4 °C, 10 min), washed once with TE buffer (10 mM Tris-HCl, 1 mM EDTA, pH 8.0, 4000× *g*, 4 °C, 10 min), and resuspended in 1 mL of the same buffer. Cell disruption was carried out on ice using a supersonic homogenizer (HD 2070, Bandelin Electronic, Berlin, Germany) with two 30 s pulses at 40% amplitude, separated by a 30 s cooling interval. Lysates were then centrifuged (10,000× *g*, 4 °C, 15 min), and the clarified supernatant was transferred into 1.5 mL reaction tubes. Samples can be stored for 1 month at −80 °C.

Glutathione peroxidase activity was quantified using the Cayman Chemical Glutathione Peroxidase Assay Kit (Item No. 703102, Ann Arbor, MI, USA) following the manufacturer’s instructions (Table 2). A positive control containing GPX was provided in the kit, and untreated bacteria served as negative controls.

Sample cells were incubated with 1 mg/L CHL. Light-treated samples as well as light-exposed controls without CHL were illuminated with red light (15.3 µmol photons·m^−2^·s^−1^) for 5 s, while dark samples and dark negative controls were wrapped in aluminum foil to protect them from light. Absorption changes were determined by measuring the change in absorbance at 340 nm over time using a microplate reader (Infinite 200 PRO, Tecan, Männedorf, Switzerland).

GPX activity was calculated from the slope of absorption changes at 340 nm per min, corrected by the background signal. Using the molar extinction coefficient of NADPH (ε = 0.00373 µM^−1^ at a path length of 6 mm, corresponding to the microplate well geometry), the following equation was used to calculate GPX activity:

$$GPX\;activity\;\left(\frac{\frac{nmol}{min}}{mL}\right)=\frac{∆A_{340}/min}{0.00373\;{µM}^{-1}}*\frac{0.19\;mL}{0.02\;mL}*dilution$$

### 2.12. Determination of Catalase (CAT) Activity

Catalase activity was quantified based on the methanol-dependent conversion of hydrogen peroxide (H_2_O_2_) to formaldehyde. The reaction was detected colorimetrically at 540 nm using a chromogenic reagent (Purpald^®^), which forms a purple bicyclic heterocycle upon oxidation by aldehydes, causing an oxidation-induced color change from colorlessness to purple. The determined CAT activity indicates the amount of enzyme that forms 1.0 nmol of formaldehyde per minute at 25 °C per unit.

Cell lysates were prepared as described in Section 2.11 (Glutathione Peroxidase Assay). Catalase activity was determined using a Catalase Assay Kit (Item No. 707002, Cayman Chemical, Ann Arbor, MI, USA) according to the manufacturer’s instructions. The total reaction volume in each well of a 96-microtiter plate was 200 µL per sample (100 µL catalase assay buffer, 30 µL MeOH, 20 µL sample or standard, respectively, 20 µL hydrogen peroxide (final concentration: 25.28 nM)).

In principle, the reaction of the assay resulted in catalase-dependent formation of formaldehyde, which was stained using 30 µL KOH (10 M) for reaction stop, followed by the sequential addition of 30 µL Purpald^®^ reagent (supplied with the Catalase Assay Kit) and (after a 10 min incubation) 10 µL potassium periodate (oxidizing agent). All steps were carried out on ice to minimize enzyme degradation.

Formaldehyde standards were prepared from the stock solution provided in the kit and diluted in assay buffer to the following final concentrations: 0.5, 15, 30, 45, 60, and 75 µM. Absorbance at 540 nm was measured using a microplate reader (Infinite 200 PRO, Tecan, Männedorf, Switzerland).

For evaluation, the means of corresponding replicates were calculated and corrected using the background signal. The slope of the calibration line (formaldehyde standards) was employed to calculate the formaldehyde concentration in the samples according to the following formula:

$$formaldehyde\;\left(µM\right)=\left(\frac{A_{540\;sample-background}}{slope}\right)*\frac{0.17\;mL}{0.02\;mL}$$

Using formaldehyde concentration, CAT-activity was determined according to following formula:

$$CAT-activity=\frac{sample\;\left[µM\right]}{20\;min}*sample\;dilution=\frac{nmol}{min}/mL$$

### 2.13. Determination of Superoxide Dismutase (SOD) Activity

Superoxide dismutase (SOD) catalyzes the dismutation of superoxide radicals into hydrogen peroxide and molecular oxygen [26]. It plays a critical role in antioxidant defense systems and is widely conserved across prokaryotic and eukaryotic organisms [26]. The enzymes are divided into four families based on their metal cofactors, consisting of copper/zinc, nickel, manganese, or iron [27]. Manganese-containing SODs are commonly found in bacteria [26].

SOD-activity was determined with the AmpliteTM Colometric Superoxide Dismutase Assay Kit (AAT Bioquest, Pleasanton, CA, USA). SOD activity was measured colorimetrically using a standard curve. The assay relies on the xanthine/xanthine oxidase system to generate superoxide radicals, which react with the chromogenic probe ReadiView™ SOD560 (supplied with the SOD-Assay), leading to a reduction in absorbance at 560 nm. The activity of the samples can be calculated using the equation of the standard curve due to the proportionality of the absorption reduction to SOD activity.

Following this, standards were prepared from an SOD standard stock solution in U/min: 100, 10, 3.33, 1.11, 0.37, 0.12, and 0.04 U/min. The color reaction, which is due to SOD-dependent xanthin-oxidation, was measured with a plate reader (Infinite 200 PRO, TECAN, Männedorf, Switzerland) at 560 nm. Cell lysates were prepared as described in Section 2.11. For data evaluation, a logistic regression formula was calculated from the standards using software provided by the following manufacturer (AAT Bioquest, Inc., Pleasanton, CA, USA). Using this formula, SOD-activity values of the samples were calculated.

### 2.14. Statistical Analysis

Statistical analyses were performed using GraphPad Prism (version 10.1.0; GraphPad Software, Boston, MA, USA; www.graphpad.com). For each experimental group, data are shown as mean ± standard deviation (SD) of biological triplicates (n = 3). Outliers were detected and removed using the ROUT method with a false discovery rate (Q) of 1%.

For comparisons between treatment groups at individual time points, unpaired *t*-tests were used. In cases where multiple comparisons were performed (e.g., across time points), the *p*-value threshold was set to 0.05, without adjustment for multiple testing unless stated otherwise. A *p*-value < 0.05 was considered statistically significant. For comparisons between two groups at individual time points (e.g., CHL^+^ vs. CHL^−^), unpaired *t*-tests were used, typically across multiple time points or conditions. For multiple comparisons against a control group (e.g., gene expression or enzyme activity), one-way ANOVA was applied, followed by Dunnett’s or the Holm–Šídák multiple comparison test, as appropriate. The threshold for statistical significance was set at α = 0.05. Where multiple comparisons were made, correction methods were applied as specified in the figure legends.

## 3. Results

### 3.1. Effect of Chlorophyllin as Function of Light on Growth of B. subtilis

The growth of the Gram-positive bacterial strain *B. subtilis* was measured over a period of 20 h, at time intervals of 20 min by optical density determination. To investigate the influence of chlorophyllin and light exposure, four different conditions were chosen for the bacteria.

- LB medium, exposed to red light for 1 min.
- 1 mg/L CHL conditioning medium, exposed to red light for 1 min.
- LB medium, protected from light.
- 1 mg/L CHL conditioning medium, protected from light.

**Figure 2:**
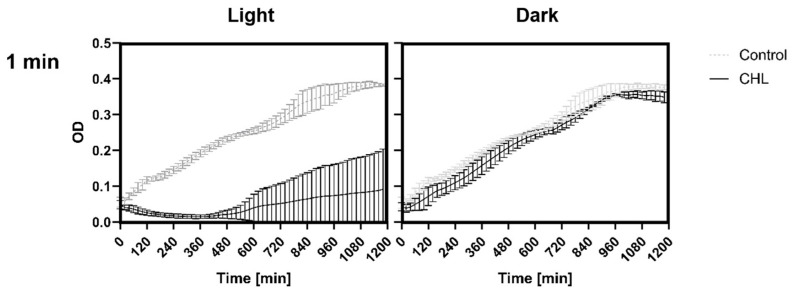
Effect of chlorophyllin and influence of light exposure on bacterial growth. *B. subtilis* was incubated during the exponential phase with 1 mg/L CHL or in LB medium (control). Light exposure for one minute (**left**) and absence of light (**right**). Optical density was measured every 20 min for a period of 20 h. The data consist of three replicates (n = 3) of the experiment, using the average from each replicate of three technical replicates, including their standard deviation.

**Figure 3:**
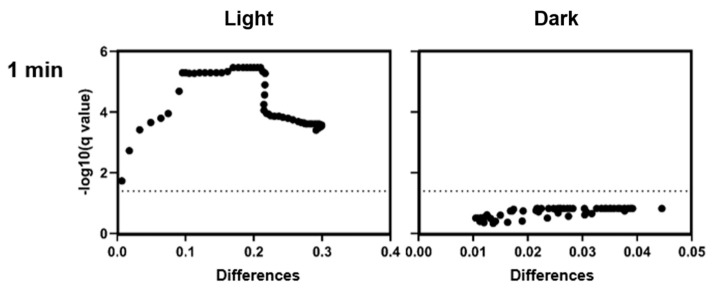
Volcano plots for the measurement data from Figure 2. One-minute exposure resulted in a statistically significant reduction in growth rate after CHL treatment at each measurement time (**left**), while lack of exposure despite CHL treatment had no statistically significant effect on growth (**right**). Each data point represents the mean difference and corresponding −log_10_(*p*-value) from unpaired *t*-tests. The horizontal line indicates the significance threshold at *p* = 0.05. No correction for multiple comparisons was applied.

A one-minute incubation under red light in the presence of CHL resulted in a significant suppression of bacterial growth compared to the control without chlorophyllin (Figure 2 left, Figure 3 left). There was a decrease in OD up to a maximum of 30% over a period of 6 h until it gradually increased again. The doubling time of the bacteria exposed to light was 40 min in the control, while the bacteria treated with CHL decreased to 186 min. For chlorophyll exposure in the dark, no significant effects on growth were observed (Figure 2 right, Figure 3 right).

### 3.2. Reduction in Survival Rate of B. subtilis After Incubation with CHL and Light Exposure

**Figure 4:**
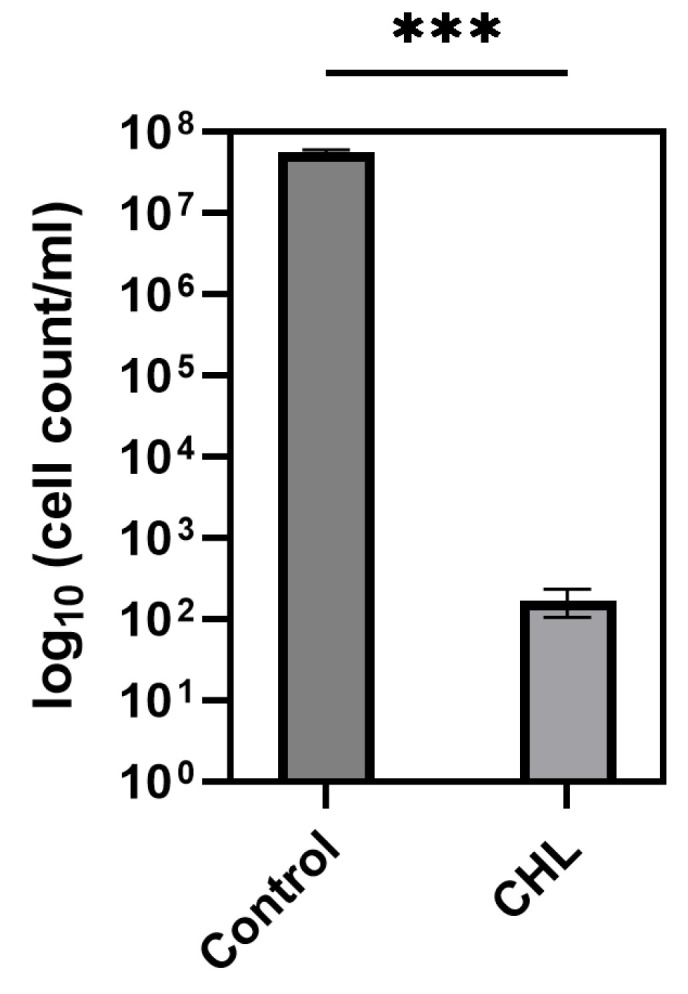
Effect of treatment of *B. subtilis* with chlorophyllin (CHL) 1 mg/L and red light 899 µmol photons·m^2^·s^−1^ for 1 min on the survival rate of *B. subtilis*. Control: light control without CHL; CHL: cells incubated with chlorophyllin during illumination. ***: *p*-value < 0.001.

Incubation of *B. subtilis* in 1 mg/L CHL and subsequent illumination with 899 µmol photons·m^−2^·s^−1^ of red light for 1 min resulted in a log5 reduction (>99.99%) in the bacteria population. Cell concentration of vital cells in the controls was about 5.7 × 10^7^ cells/mL, while after treatment, only 171 ± 65 cell/mL were detected (Figure 4).

### 3.3. Effect of Chlorophyllin and Light on Growth of B. subtilis Spores

As in the experiments with viable cells, spores were incubated in chlorophyllin for defined time intervals, then washed and transferred into fresh medium to eliminate chlorophyllin effects during subsequent incubation.

**Figure 5:**
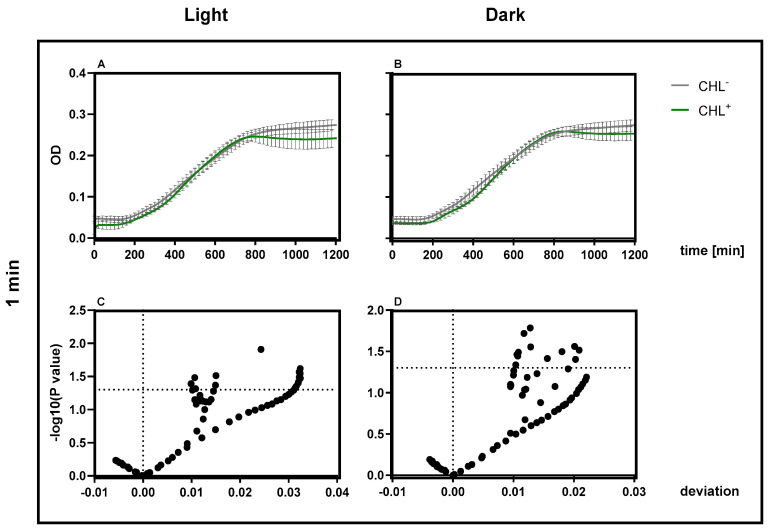
Effect of chlorophyllin (1 mg/L) and (**A**) light (899 µmol photons·m^2^·s^−1^ for 1 min) or in (**B**) darkness, respectively, on germination and growth of *B. subtilis* spores. Statistical comparison between groups was performed using unpaired *t*-tests at individual time points (**C**,**D**). Significance was defined as *p* < 0.05. Data are shown as mean ± SD.

Chlorophyllin/light treatment, performed under the same conditions as for viable *Bacillus subtilis* cells (chlorophyllin 1 mg/L and red light 899 µmol photons·m^2^·s^−1^ for 1 min, total DPI 0.14 mol/m^2^, dose = 17.73 kJ/m^2^), also resulted in weak but significant inhibition of *B. subtilis* spores (Figure 5).

**Figure 6:**
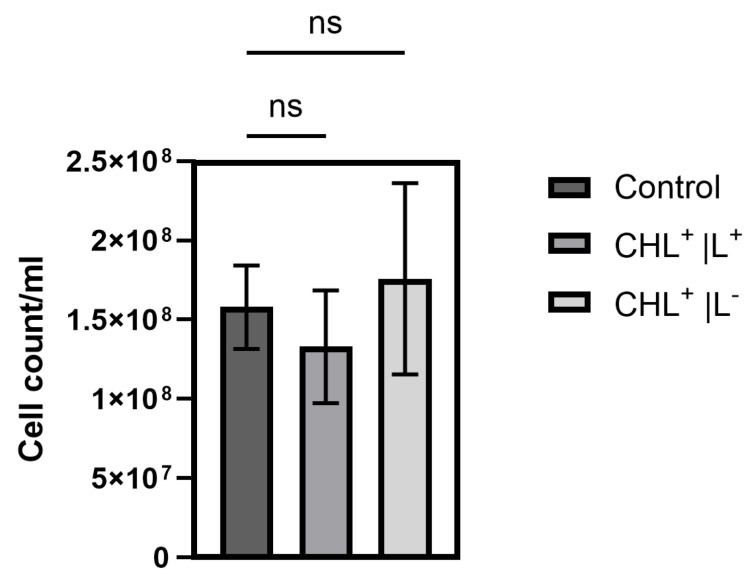
CFUs after plating CHL-incubated cells after light exposure (899 µmol photons·m^2^·s^−1^ for 1 min, DPI 0.14 mol/m^2^) or darkness, respectively. Control: Cells in absence of light and no CHL treatment. CHL^+^/L^+^: Cells incubated in CHL in presence of light. CHL^+^/L^−^: Cells with CHL incubated in darkness (ns: not significant).

In contrast to viable cells, chlorophyllin incubation and light treatment did not lead to a significant reduction in cell titer (Figure 6).

**Figure 7:**
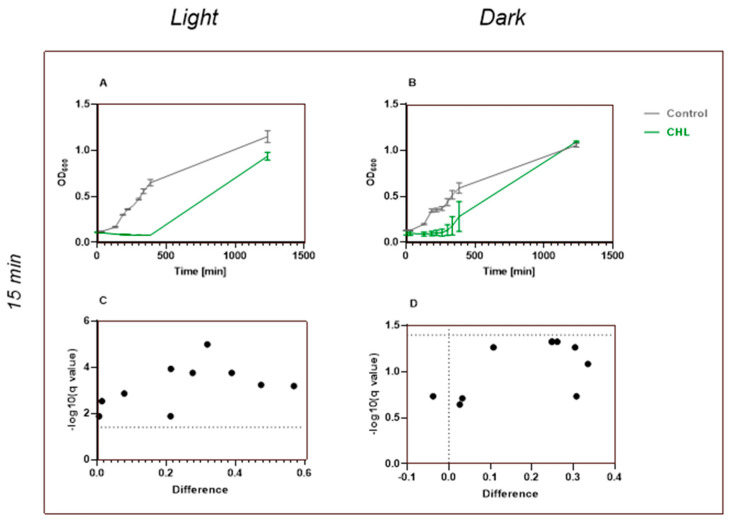
Effect of chlorophyllin and influence of light exposure on bacterial growth. *B. subtilis* with spores was incubated with 10 mg/L CHL or in LB medium (control). (**A**) Light exposure for one minute; (**B**) darkened for fifteen minutes. Optical density was measured every 30 min over several hours. Data consist of two replicates (n = 2) of experiment, using average from each replicate of two technical replicates, including their standard deviation. (**C**) One-minute exposure resulted in statistically significant reduction in growth rate after CHL treatment at most measurement times; (**D**) data set of chlorophyllin-treated bacteria under dark conditions shows no significant differences; statistical comparison between groups was performed using unpaired *t*-tests at individual time points. Significance was defined as *p* < 0.05. Data are shown as mean ± SD.

More pronounced inhibitory effects on *B. subtilis*-spores were observed with increased chlorophyllin concentrations and exposure times (15 min, radiation dose: 266.95 kJ/m^2^). After 15 min of incubation in 10 µg/mL chlorophyllin, no increase in optical density at 600 nm was observed in illuminated spore samples within the first 6 h 25 min (Figure 7A). After 24 h, growth similar to untreated controls was detected. Spores incubated with 10 µg/mL chlorophyllin in the dark started to recover after approximately 5 h and reached full culture growth by 24 h (Figure 7B).

**Figure 8:**
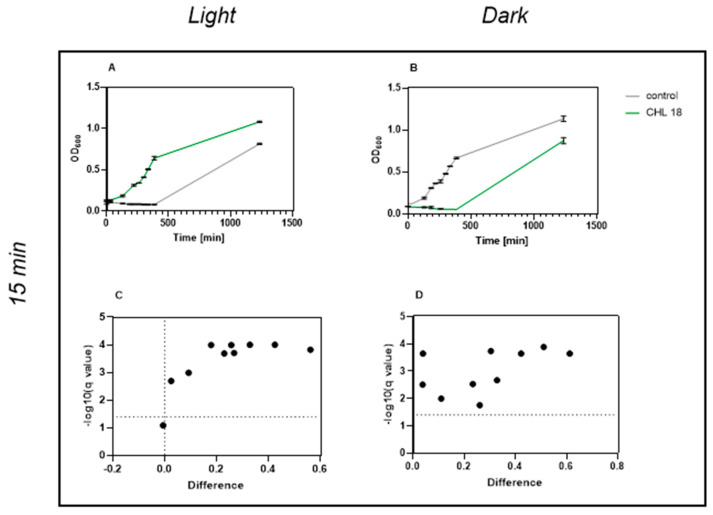
Effect of chlorophyllin (20 mg/L) and light exposure on growth of spore-containing *B. subtilis*; (**A**) growth after 15 min light exposure; (**B**) growth after 15 min in darkness; (**C**) volcano plot of data from light-exposed bacteria: light-activated Chlorophyllin exerts significant effect on growth; (**D**) statistical comparison between groups was performed using unpaired *t*-tests at individual time points. Significance was defined as *p* < 0.05. Data are shown as mean ± SD.

When spores were incubated in the presence of 20 µg/mL chlorophyllin, no cell growth was observed within 6 h 25 min in either light-exposed or dark-treated samples. However, viable cell cultures were present in both conditions after 24 h of incubation (Figure 8). Notably, chlorophyllin also affected bacterial growth in the absence of light, emphasizing the multifaceted nature of its activity.

Additional measurements were performed independently and at different time points (Supplementary Figures S1 and S2), confirming the stronger inhibitory effect of chlorophyllin under prolonged exposure and higher concentrations. These findings support its efficacy both under red light irradiation and in its absence.

### 3.4. Results of qPCR Determination of ROS-Relevant Genes

**Figure 9:**
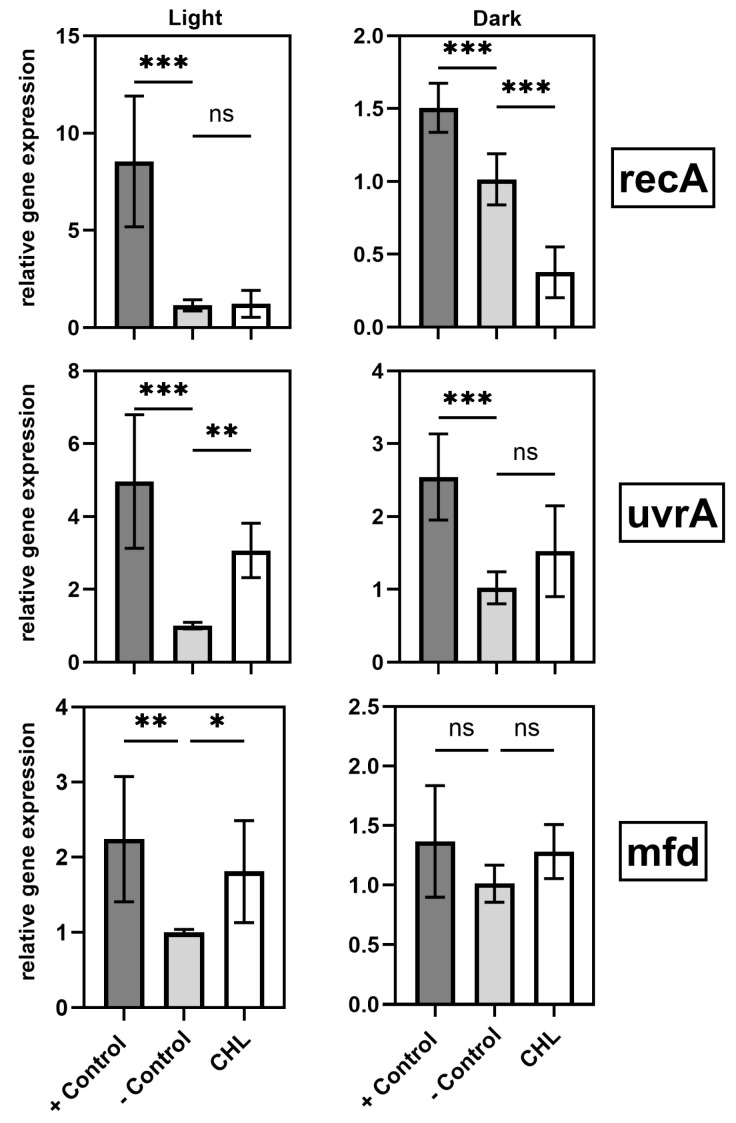
Effect of chlorophyllin (CHL) and light (15.3 µmol photons·m^2^·s^−1^) on transcription level of ROS-related genes *recA*, *uvrA*, and *mfd*, respectively. Data show relative expression compared to *gyrA* as reference gene. Left column: light-treated samples; right column: dark exposed samples. +control: positive control with 150 µM H_2_O_2_; −control: negative control: untreated cells; CHL: cells incubated in presence of chlorophyllin. N = 3, with each 3 technical triplicates. Statistical analysis performed with unifactorial ANOVA with Holm–Šídák correction (ns: not significant, *: *p* < 0.033, ** *p* < 0.002, *** *p* < 0.001).

In incubated cells with the presence of CHL under light, *recA* expression was not affected, while in dark-incubated cells with CHL, *recA* expression was significantly downregulated (Figure 9). The *uvrA* gene, which codes for an excision nuclease was found to be significantly upregulated in the presence of light and CHL but underwent no significant change in the absence of light. However, there is a visible trend towards an increase when CHL is present, even in the dark (Figure 9). Also, the *mfd* gene transcription significantly increased in the presence of CHL and light. A non-significant increase occurs in the dark-exposed samples (Figure 9).

### 3.5. Effect of CHL on Cellular ROS-Detoxification Systems in B. subtillis

#### 3.5.1. Chlorophyllin Increases Glutathione Peroxidase Activity

**Figure 10:**
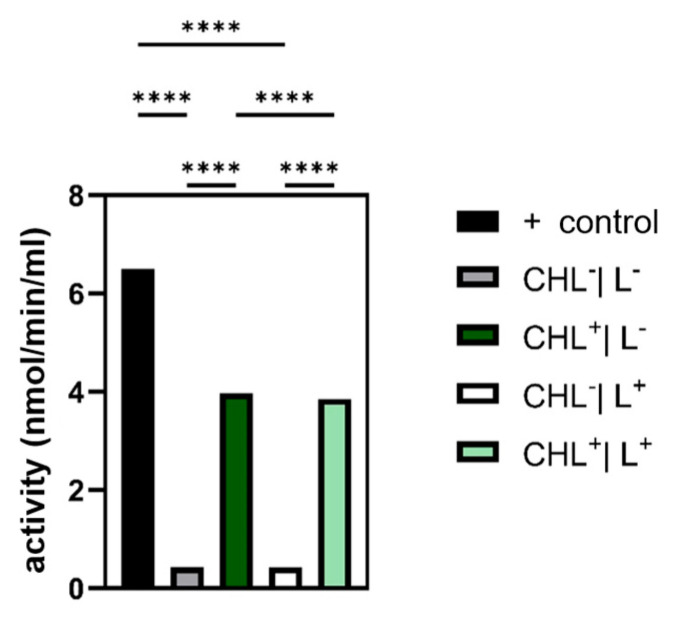
Increase in glutathione peroxidase (GPX) activity in light and darkness induced by chlorophyllin (CHL). Light alone did not alter GPX-activity. Negative controls were without CHL; light exposed samples (L^+^) were illuminated with 15.3 µmol photons·m^2^·s^−1^ of red light for 5 s. N = 4, with each 3 technical replicates. Statistical analysis performed with unifactorial ANOVA with Holm–Šídák correction. (****: *p*-value < 0.001).

It was found that addition of CHL resulted in a significant increase in GPX-activity, while light alone exhibited no effect. In darkness without CHL, the activity was 0.43 nmol/min/mL, while in the presence of CHL it was 3.97 nmol/min/mL. In light, cells without CHL showed a GPX-activity of 0.42 nmol/min/mL and with CHL an activity of 3.86 nmol/min/mL. GPX-activity in not-illuminated cells with CHL was slightly but significantly higher compared to illuminated CHL-samples (Figure 10).

#### 3.5.2. Effect of CHL and Light on Catalase (CAT)-Activity

**Figure 11:**
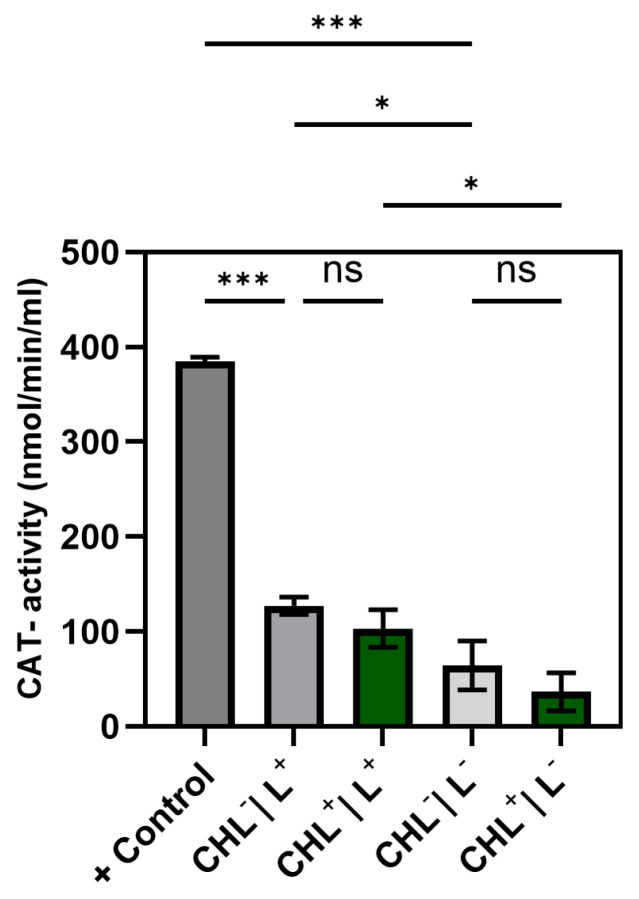
Effect of chlorophyllin (CHL) and light on catalase-activity. *B. subtilis*-cells were incubated in 1 mg/L CHL and exposed to red light (5 s, 15.3 µmol photons·m^2^·s^−1^)-CHL^+^/L^+^. Controls were dark-exposed cells with CHL (CHL^+^/L^−^), illuminated cells without CHL (CHL^−^/L^+^), dark-exposed cells with CHL (CHL^+^/L^−^), and a kit-internal positive control +control. N = 3 with 3 technical triplicates. Statistical analysis performed with unifactorial ANOVA with Holm–Šídák correction. (ns: not significant, *: *p* < 0.033, *** *p* < 0.001).

The assay did not indicate significant CHL-induced changes in CAT-activity compared to the corresponding negative controls (no CHL), neither after light exposure nor in dark-incubated cells. However, 5 s of red light (15.3 µmol photons·m^2^·s^−1^) resulted in a significant increase in CHL-treated as well as CHL-free control cells compared to their corresponding dark-exposed samples (Figure 11).

#### 3.5.3. Determination of CHL and Light-Induced Changes in Superoxide Dismutase Activity

**Figure 12:**
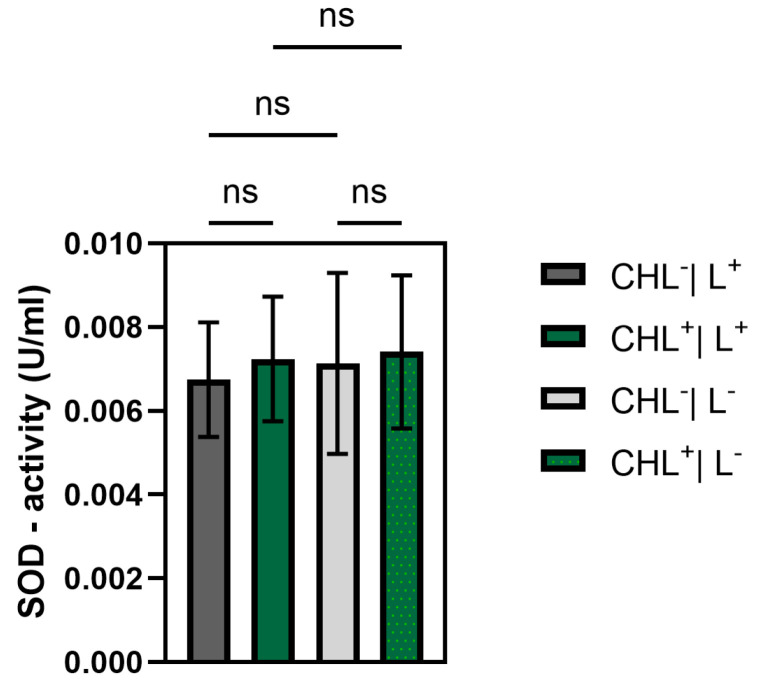
Superoxide dismutase activity is not significantly affected by chlorophyllin, 1 mg/L red light illumination (15.3 µmol photons·m^2^·s^−1^, 5 s), or combination of these factors. CHL^−^/L^+^ (no chlorophyllin/light); CHL^+^/L^+^ (with chlorophyllin/light); CHL^−^/L^−^ (no chlorophyllin/dark); CHL^+^/L^−^ (chlorophyllin/dark) (ns: not significant).

No significant differences in SOD activity were detected under the applied conditions (1 mg/L CHL, 5 s illumination with red light of 15.3 µmol photons·m^2^·s^−1^). SOD activity was the same in *B. subtilis* with or without CHL and with or without illumination, respectively (Figure 12).

## 4. Discussion

This study demonstrates that chlorophyllin-mediated photodynamic inactivation (PDI) can significantly inhibit the growth of *Bacillus subtilis* using low photosensitizer concentrations and short irradiation times. In particular, exposure to 1 mg/L chlorophyllin for 1 min resulted in several hours of growth inhibition, which compares favorably to previous studies that required higher concentrations and longer irradiation durations [25]. This rapid efficacy is of particular interest for potential applications where minimizing treatment time is critical.

Our findings align with the results of Du Lihuan et al. who investigated the direct correlation between ^1^O_2_ formation and the duration of irradiation [6]. They were able to show that increasing the irradiation duration by 15 s alone with chlorophyllin as the PS led to higher ROS formation. In this case, only a period of 0–75 s was selected with measurement intervals of ROS production of 15 s. In addition to the duration of irradiation, light intensity also influences the inactivation. It has already been described that an increase in intensity is associated with decreased growth and that longer exposure durations are necessary when the intensity is reduced [6,24].

In studies investigating the dependence on CHL concentration, Krüger et al. irradiated bacteria for 24 h and examined the concentration of CHL at which growth was affected [24]. The lowest concentration they used that showed an effect was 10 mg/L. We were able to show that even 1 mg/L and an irradiation time of 1 min were sufficient for a growth reduction in *B. subtilis*.

Although we were unable to statistically demonstrate a dark effect due to the use of lower CHL concentrations, our data suggest a tendency toward antimicrobial activity in the dark samples. This observation aligns with findings by Krüger et al., who showed that chlorophyllin must possess additional antimicrobial mechanisms, as a concentration of 2.5 mg/L in the dark was sufficient to kill *B. subtilis* [24].

Another challenge in evaluating the antibacterial effect on *B. subtilis* is the rapid transition of vegetative cells into endospores under stress. In our experiments, we addressed this issue by performing spore-specific assays. Heating cell suspensions prior to plating ensured that only spores (which survive heat treatment) contributed to colony-forming unit (CFU) counts. Although our OD600 measurements could not distinguish between spores and vegetative cells, complementary CFU assays provided additional insights into the viable cell fraction.

Finally, while our study focused exclusively on *B. subtilis* to provide a clear and detailed analysis, we acknowledge that additional work is needed to explore the effect of chlorophyllin on Gram-negative bacteria.

### 4.1. Chlorophyllin—A Highly Effective Photosensitizer

Our results confirm and extend earlier findings by Krüger et al. (2019), showing that even low CHL concentrations combined with very short irradiation (1 min, 17.73 kJ/m^2^) significantly inhibit *B. subtilis* growth over time. Their study demonstrated that 0.1 mg/L CHL with 30 min of irradiation reduced viable counts via CFU analysis [24].

Besides Gram-negative bacteria, which pose an obstacle in aPDT due to their outer membrane, spores also exhibit extremely resistant behavior. The use of chlorophyllin as a PS against spores remains understudied; consequently, this topic remains underexplored in the scientific literature. A previous study on mold spores showed promising aPDT efficacy with CHL, suggesting potential beyond bacterial applications.

To the best of our knowledge, this study is the first to demonstrate effects of CHL-based aPDT on *B. subtilis* spores. Treatment with 1 mg/L CHL and 1 min of irradiation caused significant OD600 differences compared to the control during defined periods over 20 h.

It is noteworthy that the differences occur only during defined time periods rather than continuously. However, even low CHL concentrations and very short irradiation exposure showed statistically significant effects on spore reduction. When determining the bacterial count, the effect could not be demonstrated as the standard deviation (SD) was too high to demonstrate a significant effect, although the trend here also favors a reduction in spores.

The higher resistance of spores is likely the reason why treatment of vegetative *B. subtilis* cells with 1 mg/L CHL and irradiation for one minute did not result in complete inactivation of the culture, with 0.03 ± 0.0001% surviving. The significant increase in the doubling time of vegetative cells supports the assumption, as the spores must first re-enter the vegetative growth cycle.

### 4.2. Gene Expression and SOS Response

A central finding of this study is the light-induced upregulation of DNA repair genes (*mfd*, *uvrA*) in the presence of chlorophyllin. This response suggests activation of specific DNA repair pathways triggered by ROS.

These findings underscore the complex interplay between oxidative stress and DNA repair mechanisms during photodynamic treatment. The *mfd* gene encodes a key transcription-coupled repair factor that removes stalled RNA polymerases, allowing the NER (nucleotide excision repair) machinery to access damaged DNA [21,28].

While studies have already confirmed that *mfd* in *B. subtilis* initiates transcription-coupled repair following oxidative stress, gene expression in *B. subtilis* after aPDT with CHL has not been previously studied [20]. Similarly, *uvrA*, which cooperates with *mfd* in the NER pathway, was also upregulated [19]. These results support the conclusion that chlorophyllin-mediated aPDT induces ROS, causing DNA damage that activates repair mechanisms.

In contrast, *recA*—a key regulator of homologous recombination and the SOS response [29,30]—was downregulated under dark conditions. This suggests that the observed DNA damage is sufficiently mild to be resolved by NER without full SOS activation [31,32,33]. The selective upregulation of *uvrA* may reflect differential induction thresholds among SOS genes, with *recA* responding only to more severe damage.

Another possible explanation is that different SOS genes often differ in induction thresholds and promoter affinities [34,35]. The strong upregulation of *uvrA* may reflect its sensitivity to moderate DNA damage, while *recA* requires more severe stress or full SOS activation for induction. *recA* suppression in the dark may reflect an energy-conserving strategy, possibly via enhanced *LexA* repression, to avoid unnecessary repair activation [36].

### 4.3. Intrinsic Neutralization of ROS

Given that photodynamic therapy relies on ROS generation, analyzing the cellular antioxidant defense systems—namely, catalase, glutathione peroxidase, and superoxide dismutase—was essential. In the case of glutathione peroxidase, the expectation was confirmed that bacteria treated with CHL showed a significantly increased activity compared to the control without CHL.

Although ROS were not measured directly, a significant increase in GPX activity under CHL and light treatment indicates oxidative stress involvement. In contrast, catalase activity remained unchanged, while differences between light and dark controls suggest that irradiation alone may induce moderate ROS formation. The unchanged superoxide dismutase activity underlines the selective activation of GPX and suggests that irradiation primarily leads to hydrogen peroxide formation rather than superoxide radicals.

In line with these findings, the strong downregulation of recA in dark samples likely reflects the absence of ROS-induced DNA damage and, consequently, a reduced need for SOS response activation. Under such conditions, recA repression may serve as an energy-conserving strategy, possibly supported by LexA-mediated transcriptional suppression.

### 4.4. Bifunctionality of Chlorophyllin

Our findings demonstrate that chlorophyllin not only is effective as a photosensitizer under light, but also exhibits limited antimicrobial activity in darkness. This dark effect—reflected by significant growth inhibition even without light exposure—may be attributed to non-photoactivated oxidative processes, membrane interactions, or an as-yet unexplored metabolic disruption, as suggested by increased glutathione peroxidase activity and earlier studies [24,37,38].

The full antibacterial effect requires photodynamically induced ROS formation, activating DNA repair and antioxidant defense mechanisms. The rapid inactivation of B. subtilis at low concentrations and with brief irradiation suggests promising potential for applications in food safety, hygiene, and wound treatment. Future studies should focus not only on further optimization of treatment parameters, but also on elucidating the mechanisms of light-independent effects and evaluating applicability to Gram-negative bacteria.

Based on these findings, the mechanism was further investigated. A concentration of only 10 mg/L applied for 60 min was sufficient to induce a statistically significant inhibition of bacterial growth lasting for over 6 h. Even with 1 mg/L CHL and 1 min irradiation, OD600 differences in *B. subtilis* spores were significant, indicating dark activity at low doses. Since ROS production, initiated by irradiation, is absent, we examined how gene expression levels were affected. Since *B. subtilis* is particularly sensitive in the dark, it was utilized as the subject of investigation. The expression of *uvrA* and *mfd* remained unchanged, suggesting that the NER pathway is not activated beyond physiological levels. This raises the question of whether chlorophyllin acts as a repressor or if DNA damage is simply absent.

Interestingly, *recA* was significantly downregulated, possibly as an energy-conserving survival strategy or due to LexA-mediated repression. Despite the absence of light-induced oxidative stress, glutathione peroxidase activity was significantly elevated, suggesting that chlorophyllin alone promotes organic peroxide accumulation.

## 5. Conclusions and Outlook

Our findings demonstrate that chlorophyllin is a promising antibacterial photosensitizer capable of rapidly and effectively inactivating *B. subtilis* at low concentrations and with minimal irradiation times. Future research should focus on optimizing treatment parameters—including light intensity, duration, and photosensitizer concentration—and further elucidating the molecular mechanisms underlying the differential SOS response.

Although this study focused on *B. subtilis*, the mechanisms of chlorophyllin under dark conditions and its effects on Gram-negative bacteria warrant further investigation, especially since previous work suggests additional, light-independent antimicrobial pathways and highlights the role of outer membranes in Gram-negative resistance.

Extending these investigations to include Gram-negative pathogens and assessing potential cytotoxicity on human cells will be crucial for advancing the clinical application of chlorophyllin-mediated photodynamic therapy. Notably, the observed antibacterial effect in the absence of light makes chlorophyllin a bifunctional agent with great potential in hygiene, the food industry, and superficial wound treatment.

## Figures and Tables

**Table 1:** Primers of ROS-relevant genes in *B. subtilis* [25].

Gene	Sequence (5′-3′)	Melting Temperature (°C)	Length of Amplicon (bp)
*gyrA* (housekeeping gene)	CAAACATTCCTCCGCACCAG	59.48	143
	GCTGCGTCCCAAGATTTGAC	59.83	
*recA*	GGCTCCCTCGCTCTTGATAC	59.97	179
	ACCGGATCTAACGCATGCTC	60.25	
*uvrA*	GCCGGATGTGGATGCAATTG	60.25	157
	TTCCGGACAATGAGGCTTCC	60.04	
*mfd*	AGCCGGGAATCTTCTTGGTG	60.04	123
	TGCTCTGTCTTCGCTGTGTC	60.32	

**Table 2:** Reaction mix for determination of GPX activity.

Reaction/Well	Assay-Buffer (µL)	Co-Substrate (µL)	NADPH (µL)	GPX (µL)	Sample (µL)
Background	70	50	50	-	-
Positive control	50	50	50	20	-
Sample	50	50	50	-	20

## Data Availability

The original contributions presented in this study are included in the article/Supplementary Materials. Further inquiries can be directed to the corresponding author.

## Supplementary Materials

The following supporting information can be downloaded at: https://www.mdpi.com/article/10.3390/microorganisms13061189/s1, Figure S1: Data from different measurements under (A,C,E) 1-min light exposure or (B,D,F) 1-min darkness with 1 µg/mL Chlorophyllin addition are illustrated; Figure S2: Data from different measurements under (A,C,E) 15-min light exposure or (B,D,F) 15-min darkness with 20 µg/mL Chlorophyllin addition are illustrated.
