# Supplementary material for: Chlorophyllin-Mediated Photodynamic Inactivation: Dosage and Time Dependency in the Inhibition of Bacillus subtilis
Source: Microorganisms. 2025 May 23;13(6):1189. doi: 10.3390/microorganisms13061189 (PMC12195115; doi:10.3390/microorganisms13061189)
Supplement: Supplementary file 1 [file microorganisms-13-01189-s001.zip › microorganisms-3517215-supplementary.pdf]

**DATA FOR SUPPLEMENTS

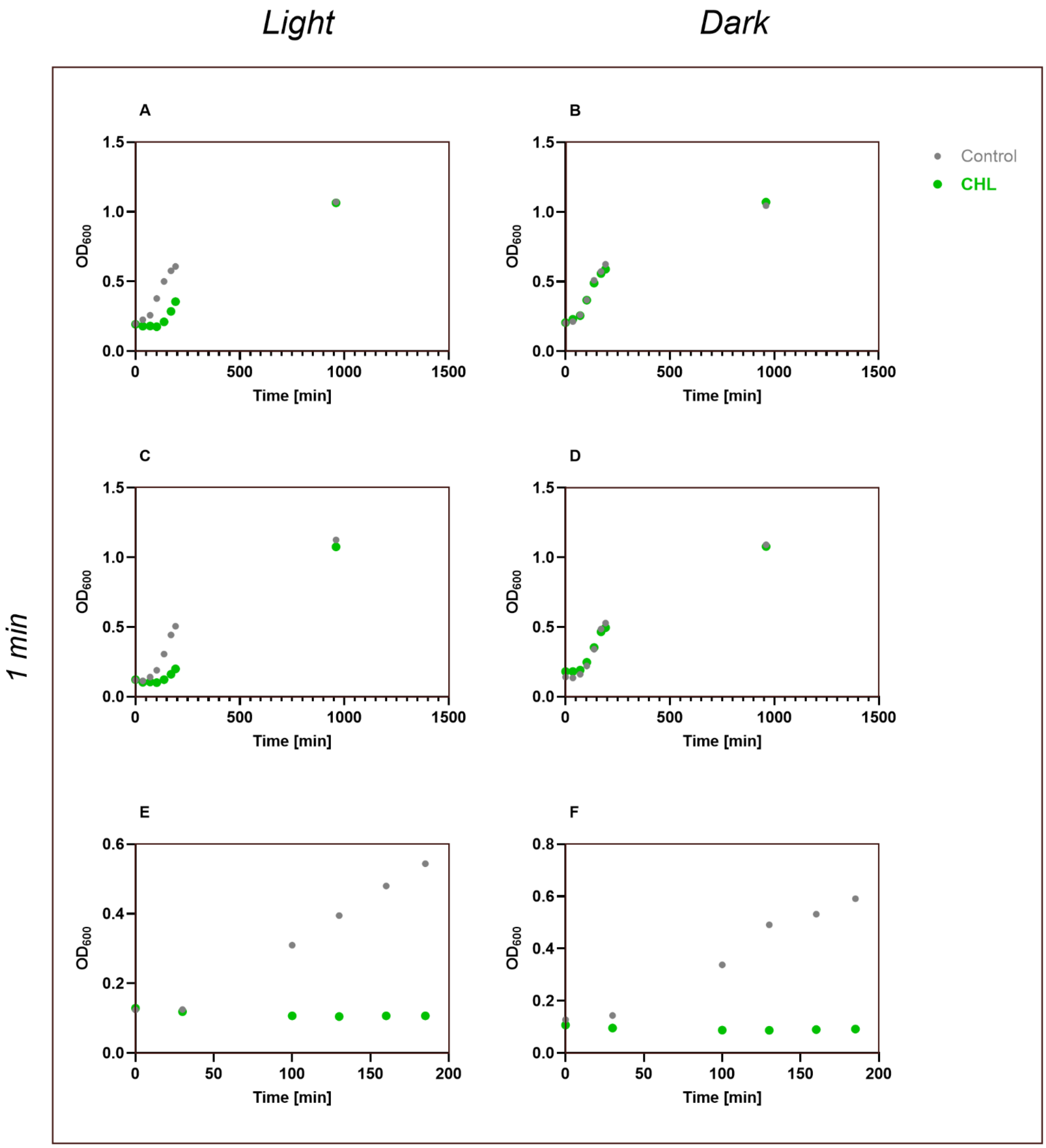

Figure S1. Data from different measurements under (A,C,E) 1-min light exposure or (B,D,F) 1-min darkness with 1 μg/mL Chlorophyllin addition are illustrated.

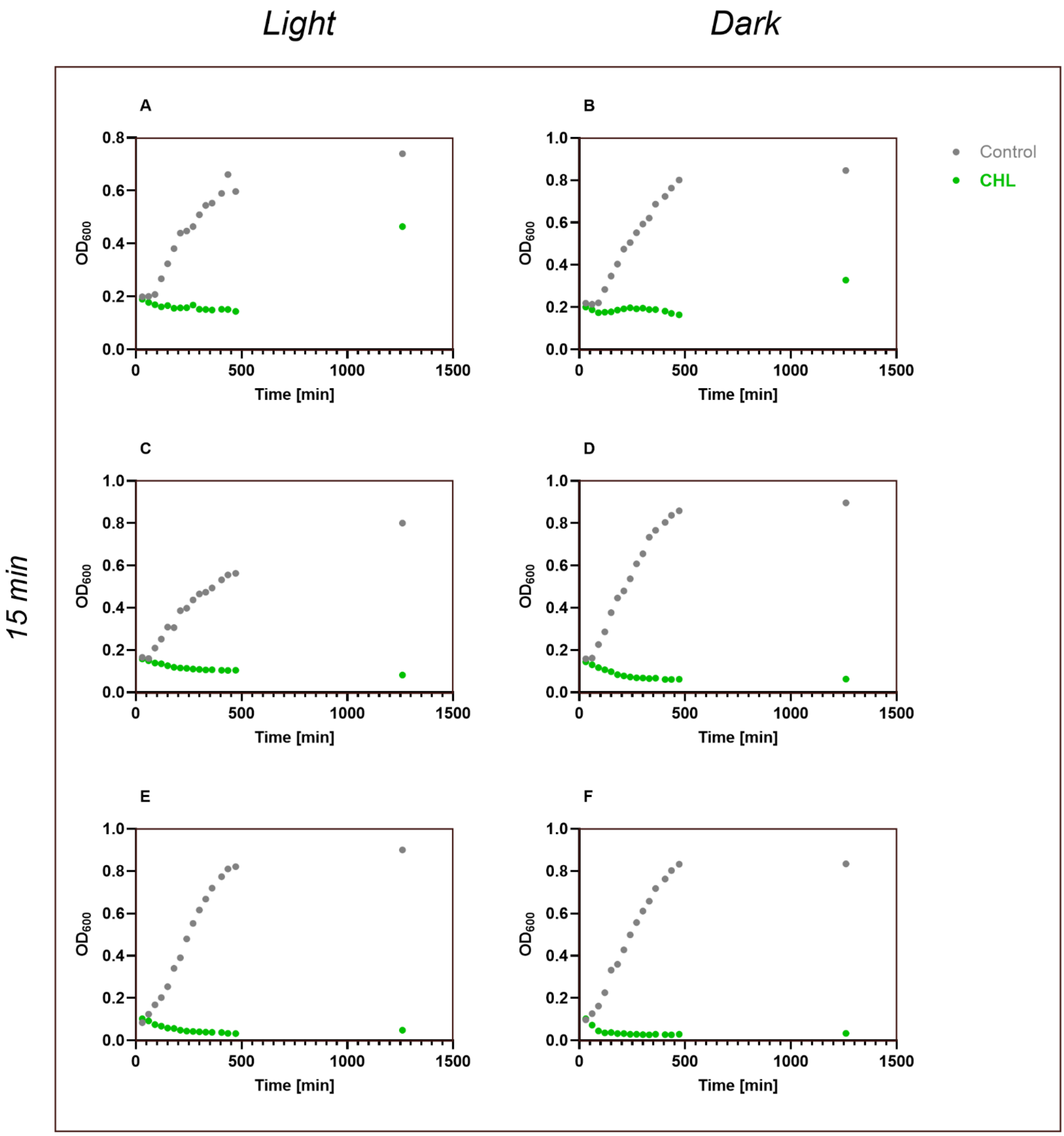

Figure S2. Data from different measurements under (A,C,E) 15-min light exposure or (B,D,F) 15-min darkness with 20 μg/mL Chlorophyllin addition are illustrated.
